# Supplementary material for: Pulsed Electric Fields to Improve the Use of Non-Saccharomyces Starters in Red Wines
Source: Foods. 2021 Jun 25;10(7):1472. doi: 10.3390/foods10071472 (PMC8304018; doi:10.3390/foods10071472)
Supplement: Supplementary file 1 [file foods-10-01472-s001.zip › foods-1250291-supplementary.pdf]

Supplementary Materials

Table S1. Results of the sensory analysis of the wines. Values are means ± sd (n=3). Values with the same letter or without * in the same column are not significantly different (p<0.05).

Yeasts/ Parameters	Colour intensity	Hue	Turbidity	Aromatic intensity	Aromatic quality	Herbaceous	Floral	Fruity	Reduction	Oxidation	Body	Sweetness	Acidity	Global perception
Sc Untreated	3.56 ± 0.73 β	2.33 ± 0.50 α	1.33 ± 0.50 α	3.22 ± 0.67 α	2.56 ± 0.88 α	1.78 ± 0.83 α	1.67 ± 1.12 α	2.56 ± 1.01 αβ	1.67 ± 1.12 α	2.44 ± 1.13 β	2.89 ± 0.60 α	1.67 ± 0.71 α	3.00 ± 1.00 βγ	2.56 ± 1.01 α
Lt Untreated	3.22 ± 0.97 α	2.44 ± 0.53 α	1.33 ± 0.50 α	3.00 ± 0.71 α	3.11 ± 0.93 α	1.44 ± 0.53 α	1.89 ± 0.93 α	2.67 ± 1.00 β	1.22 ± 0.44 α	1.78 ± 1.09 α	2.44 ± 0.73 α	1.89 ± 1.05 αβ	3.56 ± 0.88 γ	2.89 ± 1.05 α
Hv Untreated	3.11 ± 0.78 αβ	2.78 ± 0.97 α	1.56 ± 0.73 α	3.22 ± 0.67 α	3.00 ± 0.71 α	1.56 ± 0.73 α	1.67 ± 0.87 α	2.00 ± 0.50 αβ	1.78 ± 0.97 α	1.56 ± 0.73 α	2.67 ± 0.87 α	2.00 ± 1.00 β	2.78 ± 0.83 αβ	2.78 ± 0.67 α
Td Untreated	3.11 ± 0.78 α	2.22 ± 1.09 α	1.33 ± 0.50 α	2.56 ± 0.73 α	3.11 ± 0.78 α	1.56 ± 0.73 α	2.33 ± 0.87 α	2.33 ± 1.22 αβ	1.33 ± 0.71 α	1.78 ± 0.83 α	2.56 ± 0.53 α	1.56 ± 0.73 α	3.00 ± 0.71 αβ	2.67 ± 0.87 α
Mp Untreated	3.11 ± 0.78 α	2.11 ± 0.78 α	1.22 ± 0.44 α	2.78 ± 0.83 α	2.67 ± 0.71 α	1.44 ± 0.53 α	1.89 ± 1.05 α	1.67 ± 0.87 α	1.22 ± 0.67 α	1.56 ± 0.73 α	2.22 ± 0.83 α	1.67 ± 0.87 α	2.89 ± 1.27 αβ	2.22 ± 0.67 α
Sp Untreated	3.11 ± 0.78 αβ	2.56 ± 0.73* α	1.33 ± 0.71 α	2.78 ± 0.83 α	2.56 ± 0.73 α	1.56 ± 0.88 α	1.78 ± 0.67 α	2.00 ± 0.71* αβ	1.56 ± 1.01 α	1.44 ± 0.73 α	2.56 ± 0.88 α	1.67 ± 0.87 α	2.56 ± 1.33 α	2.44 ± 0.53 α
Sc PEF	4.00 ± 0.71 β	2.78 ± 0.83 α	1.33 ± 0.50 α	3.00 ± 0.50 α	3.11 ± 0.60 α	1.67 ± 1.00 α	1.56 ± 1.01 α	2.56 ± 0.88 αβ	1.56 ± 1.01 α	2.22 ± 0.83 β	2.67 ± 0.71 α	1.44 ± 0.73 α	3.44 ± 1.13 βγ	3.00 ± 0.50 α
Lt PEF	3.22 ± 0.83 α	2.11 ± 0.78 α	1.33 ± 0.50 α	2.78 ± 0.83 α	3.33 ± 0.87 α	1.56 ± 1.13 α	1.89 ± 0.93 α	2.78 ± 0.67 β	1.44 ± 0.73 α	1.67 ± 0.87 α	2.67 ± 0.87 α	2.00 ± 1.12 αβ	4.00 ± 0.50 γ	3.00 ± 1.00 α
Hv PEF	3.22 ± 0.88 αβ	2.44 ± 0.88 α	1.33 ± 1.09 α	3.00 ± 0.71 α	3.11 ± 0.97 α	1.44 ± 0.88 α	2.00 ± 0.71 α	2.78 ± 0.87 αβ	1.33 ± 0.88 α	1.78 ± 0.88 α	2.44 ± 0.67 α	1.78 ± 0.93 β	3.33 ± 0.67 αβ	2.89 ± 1.22 α
Td PEF	3.33 ± 0.87 α	2.44 ± 0.73 α	1.78 ± 0.97 α	2.78 ± 0.83 α	2.67 ± 0.71 α	1.67 ± 1.00 α	1.78 ± 0.67 α	2.11 ± 0.78 αβ	1.56 ± 0.88 α	1.56 ± 0.53 α	2.89 ± 0.60 α	2.11 ± 1.05 α	3.00 ± 0.71 αβ	2.78 ± 0.44 α
Mp PEF	3.22 ± 0.83 α	2.56 ± 0.73 α	1.67 ± 0.87 α	2.89 ± 0.93 α	2.89 ± 1.27 α	1.67 ± 0.87 α	1.89 ± 1.05 α	2.33 ± 1.32 α	1.11 ± 0.33 α	1.56 ± 0.73 α	2.78 ± 0.67 α	1.78 ± 0.83 α	3.11 ± 0.93 αβ	2.78 ± 0.44 α
Sp PEF	3.56 ± 0.88 αβ	1.67 ± 0.87* α	1.22 ± 0.44 α	3.33 ± 1.00 α	3.11 ± 1.54 α	1.22 ± 0.44 α	2.22 ± 1.20 α	3.11 ± 1.36* αβ	1.89 ± 1.27 α	1.00 ± 0.00 α	2.44 ± 0.53 α	2.00 ± 0.87 α	2.44 ± 1.13 α	3.22 ± 1.09 α

PEF treatment: * = significant difference between counterparts. Fermentative biotechnology: Greek letters.
